# Molecular Characteristics of Methicillin-Resistant Staphylococci Clinical Isolates from a Tertiary Hospital in Northern Thailand

**DOI:** 10.1155/2018/8457012

**Published:** 2018-11-19

**Authors:** Thawatchai Kitti, Rathanin Seng, Natnaree Saiprom, Rapee Thummeepak, Narisara Chantratita, Chalermchai Boonlao, Sutthirat Sitthisak

**Affiliations:** ^1^Faculty of Oriental Medicine, Chiang Rai College, Chiang Rai, Thailand; ^2^Department of Microbiology and Immunology, Faculty of Tropical Medicine, Mahidol University, Bangkok, Thailand; ^3^Department of Microbiology and Parasitology, Faculty of Medical Sciences, Naresuan University, Phitsanulok, Thailand; ^4^Chiangrai Prachanukroh Hospital, Amphoe Meuang, Chiangrai, Thailand; ^5^Centre of Excellence in Medical Biotechnology, Faculty of Medical Science, Naresuan University, Phitsanulok, Thailand

## Abstract

Methicillin-resistant staphylococci are now recognized as a major cause of infectious diseases, particularly in hospitals. Molecular epidemiology is important for prevention and control of infection, but little information is available regarding staphylococcal infections in Northern Thailand. In the present study, we examined antimicrobial susceptibility patterns, detection of antimicrobial resistance genes, and SCC*mec* types of methicillin-resistant *S. aureus* (MRSA) and methicillin-resistant coagulase-negative staphylococci (MR-CoNS) isolated from patients in a hospital in Northern Thailand. The species of MRSA and MR-CoNS were identified using combination methods, including PCR, MALDI-TOF-MS, and *tuf* gene sequencing. The susceptibility pattern of all isolates was determined by the disk diffusion method. Antimicrobial resistance genes, SCC*mec* types, and ST239 were characterized using single and multiplex PCR. ST239 was predominant in MRSA isolates (10/23). All MR-CoNS (*N*=31) were identified as *S. haemolyticus* (*N*=18), *S. epidermidis* (*N*=3), *S. cohnii* (*N*=3), *S. capitis* (*N*=6), and *S. hominis* (*N*=1). More than 70% of MRSA and MR-CoNS were resistant to cefoxitin, penicillin, oxacillin, erythromycin, clindamycin, gentamicin, and ciprofloxacin. In MRSA isolates, the prevalence of *erm*A (78.3%) and *erm*B (73.9%) genes was high compared to that of the *erm*C gene (4.3%). In contrast, *erm*C (87.1%) and *qac*A/B genes (70.9%) were predominant in MR-CoNS isolates. SCC*mec* type III was the dominant type of MRSA (13/23), whereas SCC*mec* type II was more present in *S. haemolyticus* (10/18). Ten MRSA isolates with SCC*mec* type III were ST239, which is the common type of MRSA in Asia. This finding provides useful information for a preventive health strategy directed against methicillin-resistant staphylococcal infections.

## 1. Introduction

*Staphylococcus* is recognized as an important cause of nosocomial infection. The most prominent pathogen of the genus is the coagulase-positive *Staphylococcus aureus*, which causes osteomyelitis, endocarditis, septic arthritis, pneumonia, and skin infections [1]. However, coagulase-negative staphylococci (CoNS) such as *S. epidermidis*, *S. haemolyticus*, *S. lugdunensis*, *S. cohnii*, *S. capitis*, and *S. hominis* are also associated with various infections with possible fatal outcomes in newborns or immunocompromised patients [2]. It is well established that staphylococcal infections in hospitals show an increasing prevalence of methicillin-resistant *S. aureus* (MRSA) and methicillin-resistant coagulase-negative staphylococci (MR-CoNS) isolates [3, 4]. Methicillin resistance in staphylococci results from the recombinase-mediated insertion of the staphylococcal chromosomal cassette *mec* (SCC*mec*), the mobile genetic element that carries *mec*A and various antibiotic resistance genes. The *mec*A gene encodes penicillin-binding protein PBP2a that has a low affinity for *β*-lactam antibiotics [5]. To date, eleven SCC*mec* types (I–XI) have been identified. SCC*mec* types I, II, and III have been associated more frequently with hospital-acquired MRSA (HA-MRSA), while SCC*mec* types IV and V are the most dominant in MRSA infections acquired in the community (CA-MRSA) [6]. Previous studies reported the prevalence rate of these major clones varies markedly in different geographic regions; the predominant HA-MRSA clone in Asian countries is MRSA-ST239-III [7]. *S. epidermidis* has been found to harbor SCC*mec* types I, II, III, IV, and V. Likewise, SCC*mec* types II, III, and V have been discovered in *S. haemolyticus* [8]. It is generally accepted that the tolerance of chlorhexidine in *S. aureus* is associated with the family of the *qac* (*qac*A/B) gene, which encodes proton-motive force-dependent export pumps [9]. Recently, a study suggested that *qac*A/B carriage might contribute to an increasing global dominance of CC22 and ST239 clones [10]. Erythromycin resistance in staphylococci is predominantly caused by erythromycin resistance RNA methylase, whose action also affects resistance to other macrolides, lincosamides, and streptogramin B (MLS_B_). This resistance is mediated by the *erm*-type genes, caused almost exclusively by *erm*A or *erm*C [11]. Little information is available on the molecular epidemiology of MRSA and MR-CoNS in Northern Thailand. This study was designed to characterize the antimicrobial resistance genes and SCC*mec* types of MRSA and MR-CoNS isolated from a hospital in Chiangrai Province located in Northern Thailand. These data will provide insights into the epidemiology of the MRSA and MR-CoNS in this region.

## 2. Materials and Methods

### 2.1. Bacterial Isolates

A total of 54 clinical isolates of staphylococci were collected from November 2015 to October 2016 from patients who were admitted to Chiangrai Prachanukroh Hospital, Chiangrai. The hospital is a (756-bed) teaching hospital that handles ∼3,500 admissions per day, located in the north of Thailand. The isolates were collected from blood (39 isolates, 72.2%), pus (10 isolates, 18.5%), sputum (4 isolates, 7.4%), and other body fluids (1 isolate, 1.9%).

The bacteria were initially identified by colony morphology, mannitol fermentation, Gram characteristics, catalase test, coagulase test, and DNase activity. The phenotypic methicillin resistance was assessed using the cefoxitin disk diffusion method in accordance with the Clinical and Laboratory Standard Institute guidelines (CLSI M100-S24) at our clinical laboratory, which has been accredited by the College of American Pathologists [12]. *S. aureus* NCTC10442, *S. aureus* JCSC10442, and *S. aureus* WIS were used as reference strains for SCC*mec* typing. *S. aureus* COL was used as a positive control for *mec*A gene detection.

### 2.2. Species Identification of Methicillin-Resistant Staphylococci

All isolates were confirmed as staphylococci by a PCR method based on the 16S rRNA gene [13]. The *mec*A gene was detected in all isolates to confirm the methicillin resistance [14]. MRSA was identified using PCR for detecting the *nuc* gene as previously described by Sasaki et al. [15]. The species level of MR-CoNS was identified by MALDI-TOF-MS [16] and *tuf* gene sequencing [17].

The direct colony of MALDI-TOF-MS analysis was analyzed as previously described [15]. The score identification criteria were used as follows: a score of 2.000 to 3.000 indicated species-level identification, a score of 1.700 to 1.999 indicated genus-level identification, and a score <1.700 indicated an unreliable identification [18].

### 2.3. Determination of Antibiotic Susceptibility

The antibiotic susceptibility patterns of bacteria to penicillin (P; 10 units), clindamycin (DA; 2 *µ*g), chloramphenicol (C; 30 *µ*g), gentamicin (CN; 10 *µ*g), erythromycin (E; 15 *µ*g), cefoxitin (FOX; 30 *µ*g), sulfamethoxazole/trimethoprim (SXT; 1.25/23.75 *µ*g), vancomycin (VA; 30 *µ*g), rifampicin (RD; 5 *µ*g), linezolid (LZD; 30 *µ*g), mupirocin (MUP; 5 *µ*g), ciprofloxacin (CIP; 5 *µ*g), fusidic acid (FD; 10 *µ*g), and novobiocin (NV; 5 *µ*g) (Oxoid) were determined according to the antibiotic disk diffusion method (CLSI, 2014).

### 2.4. Determination of SCC*mec* Types

Multiplex PCR was carried out as described by Zhang et al. [19]. Amplification was performed in a total volume of 25 *µ*l containing 3 *µ*l of 10x buffer with 15 mM of Mg^2+^, 2.5 *µ*l of 2.5 mM dNTP, 0.2 *µ*l of 5 U *Taq* polymerase, various concentrations of each primer, and 3 *µ*l of the DNA template. The condition for thermal cycler was set as follows: denaturation at 94°C for 4 min followed by 30 cycles at 94°C for 20 sec, 55°C for 30 sec, and 72°C for 30 min and a final extension at 72°C for 5 min. All PCR products were visualized using gel electrophoresis with 1% agarose gel stained with ethidium bromide.

### 2.5. ST239 Identification

The ST239 was determined by the PCR method using two oligonucleotide primer sets as previously described by Feil et al. [20]. Amplification reaction was performed with the following condition: 1 cycle of predenaturation at 95°C for 15 min followed by 30 cycles at 95°C for 30 sec, 55°C for 30 sec, and 72°C for 30 sec and a final extension at 72°C for 7 min.

### 2.6. Detection of Antibiotic and Disinfectant Resistance Genes

The other antibiotic and disinfectant resistance genes including the *erm*A, *erm*B, *erm*C, and *qac*A/B genes (disinfectant) were detected by PCR as previously described [21–23]. The primer sets are shown in Supplementary Material 1. All PCR products were visualized using gel electrophoresis with 1% agarose gel stained with ethidium bromide. The absence of bias was ensured by the sequencing of each gene in the representative isolates.

## 3. Results

### 3.1. Species Distribution of Staphylococci

**Figure 1:**
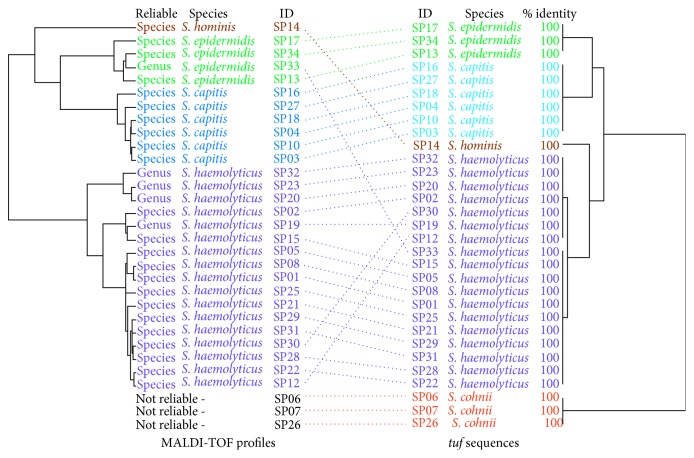
Species distribution of MR‐CoNS isolates identified by MALDI‐TOF and *tuf* gene sequencing.

The species of all isolates were identified by combined methods, including biochemical tests, PCR, MALDI-TOF-MS, and DNA sequencing. All 23 MRSA isolates were confirmed by detection of the *nuc* gene, and all species of MR-CoNS isolates were confirmed by *tuf* gene sequencing. The species distribution of MR-CoNS is given in Figure 1. The species included methicillin-resistant *S. haemolyticus* (*n*=18), methicillin-resistant *S. epidermidis* (*n*=3), methicillin-resistant *S. cohnii* (*n*=3), methicillin-resistant *S. capitis* (*n*=6), and methicillin-resistant *S. hominis* (*n*=1).

### 3.2. Antimicrobial Susceptibility Testing

**Figure 2:**
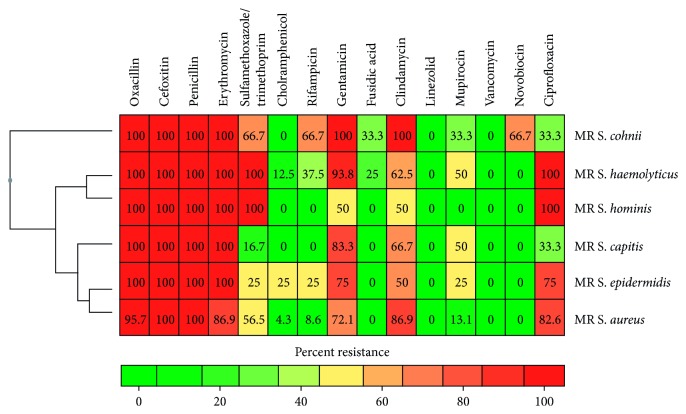
Antimicrobial resistance patterns of MRSA and MR-CoNS isolates to 15 antimicrobial agents.

All methicillin-resistant staphylococci were tested for their susceptibility against 15 commonly used antibiotics (Figure 2). All MRSA isolates were sensitive to linezolid, fusidic acid, novobiocin, and vancomycin. Prevalence of resistance among the isolates was as follows: cefoxitin (100%), penicillin (100%), oxacillin (95.7%), erythromycin (86.9%), clindamycin (86.9%), gentamicin (72.1%), ciprofloxacin (72.1%), sulfamethoxazole/trimethoprim (56.5%), mupirocin (13.1%), rifampicin (8.6%), and chloramphenicol (4.3%). Likewise, none of the MR-CoNS isolates were resistant to linezolid and vancomycin. However, prevalence of resistance among the isolates was as follows: oxacillin (100%), cefoxitin (100%), penicillin (100%), gentamicin (87.1%), erythromycin (86.9%), ciprofloxacin (77.4%), clindamycin (64.5%), sulfamethoxazole/trimethoprim (70.9%), mupirocin (41.9%), rifampicin (29.0%), fusidic acid (16.1%), chloramphenicol (9.7%), and novobiocin (6.5%).

### 3.3. Distribution of SCC*mec* Types and ST239 Type Detection

All 54 staphylococci were *mec*A-positive isolates. SCC*mec* types of all isolates were assigned by multiplex PCR according to the procedures and primer sets listed. As shown in Table 1, all MRSA isolates could be classified into six types of SCC*mec* elements: types I (*n*=6), II (*n*=1), III (*n*=13), IVa (*n*=1), IVb (*n*=1), and V (*n*=1). The distribution of SCC*mec* types in all MR-CoNS used in this study was ranked as types I (*n*=3), II (*n*=10), III (*n*=5), IVa (*n*=3), IVc (*n*=2), and V (*n*=2). SCC*mec* type II was the predominant clone (55.6%) in *S. haemolyticus*. The distribution of SCC*mec* types in each species is given in Table 1. Interestingly, using the multiplex PCR method, we could detect ST239 in 10 isolates of MRSA, and all of them were of SCC*mec* type III.

### 3.4. Disinfectant and Antibiotic Resistance Genes

Among the 54 methicillin-resistant staphylococci isolates as shown in Table 1, 28 isolates (51.9%) harbored *qac*A/B. These included 6 isolates (26.1%) of *S. aureus*, 13 isolates (72.2%) of *S. haemolyticus*, 3 isolates (100%) of *S. cohnii*, 5 isolates (83.3%) of *S. capitis*, and 1 isolate (100%) of *S. hominis*. The erythromycin resistance genes (*erm*A, *erm*B, and *erm*C) were also detected in MRSA and MR-CoNS. The prevalence of *erm*A, *erm*B, and *erm*C genes found in MRSA was 78.3% (18/23), 73.9% (17/23), and 4.3% (1/23), respectively, whereas 12.9% (4/31), 12.9% (4/31), and 87.1% (27/31) of *erm*A*, erm*B, and *erm*C, respectively, were present in MR-CoNS. The most prevalent *erm*C gene was detected in MR-CoNS, including 88.9% in *S. haemolyticus*, 100% in *S. cohnii*, 100% in *S. capitis*, and 100% in *S. hominis*. The *erm*A and *erm*B genes were found in *S. epidermidis* and *S. capitis* (Table 1).

## 4. Discussion

Methicillin-resistant staphylococci have dispersed worldwide and continue to be among the most common hospital pathogens. The prevalence and characterization of MRSA and MR-CoNS in hospitals have been reported from different parts of the world [24, 25]. However, the increase of antibiotic resistance in nosocomial isolates of MRSA and MR-CoNS aggravates this problem and poses a great challenge for the management of hospital-acquired infections. In the present study, we found that the 54 staphylococcal isolates belonged to 6 different species. The species distribution identification by MALDI-TOF-MS was consistent with the species identified by *tuf* gene sequencing, with the exception of one isolate (SP33) (Figure 1). Using MALDI-TOF-MS, this isolate was identified as *S. epidermidis*, but *tuf* gene sequencing identified it as *S. haemolyticus*. We assumed that the species assigned by *tuf* gene sequencing was more accurate because the score of MALDI-TOF-MS was only at the level of genus identification. Moreover, MALDI-TOF-MS could not identify 3 isolates of MR-CoNS. These 3 isolates were identified as *S. cohnii* by *tuf* gene sequencing. This result was consistent with a previous study reporting that MALDI-TOF-MS could not identify *S. cohnii* to the species level [26]. Additionally, a phylogenetic tree based on *tuf* gene sequencing was compared with the MALDI-TOF dendrogram for all 31 isolates of MR-CoNS (Figure 1). Interestingly, if the disagreement for one isolate (SP 33) was not considered, the structure of each species was broadly in alignment. Only *S. hominis* was located in different structures of both phylogenetic trees. To the best of our knowledge, this is the first comparison between phylogenetic tree based on *tuf* gene sequencing and MALDI-TOF dendrogram of MR-CoNS.

We found that MRSA and MR-CoNS isolates were resistant to multiple antibacterial agents (Figure 2). Among MR staphylococci isolates, 82.6% were resistant to 7–10 antibiotics (96.8% of MR-CoNS and 60.9% of MRSA). This result is similar to the findings in China and France with a high rate of antibiotic resistance within MRSA clinical isolates [27, 28]. In this study, all MRSA and MR-CoNS isolates were sensitive to vancomycin and linezolid. Thus, these drugs remain suitable options for the treatment of serious infections caused by MRSA and MR-CoNS.

The *mec*A gene, encoding a PBP variant which confers resistance to methicillin, was detected in 100% of staphylococci isolated in this study. *mec*A is carried by the mobile genetic element SCC*mec*. The distribution of different SCC*mec* types in methicillin-resistant staphylococci varied depending on the host species, bacterial clones, and possibly geographical locations [29]. SCC*mec* typing has become essential for the epidemiological characterization of MRSA and MR-CoNS clones. In this study, 54 methicillin-resistant staphylococci were investigated for their SCC*mec* types; SCC*mec* type III was found to be predominant, with a proportion of 56.5% (13/23) of MRSA isolates. Our results are in agreement with Chongtrakool et al., who reported SCC*mec* type III as the predominant type in many Asian countries such as Saudi Arabia, India, Sri Lanka, Singapore, Indonesia, Thailand, Vietnam, Philippines, and China, whereas SCC*mec* type I of MRSA isolates which shows high prevalence in Iran (56.9%) was found to be only 26.1% in our study [29, 30].

We found that ST239 was detected in 43.5% (10/23) MRSA isolates, and all positive clones carried SCC*mec* type III (ST239-SCC*mec* III). Previous studies have demonstrated that ST239-SCC*mec* III is the endemic HA-MRSA in many Asian countries, although a recent study showed that this clone is being steadily displaced by emerging CA-MRSA clones [24]. ST239-SCC*mec* III was also reported to be the dominant clone among MRSA clinical isolates in Singapore during 2006–2010. Similarly, because of its high prevalence (77.1%), ST239-SCC*mec* II was accounted for as the most dominant nosocomial MRSA clone in 18 hospitals in China [31]. It has been reported that ST239-SCC*mec* III was detected in at least 90% of HA-MRSA isolates in Sappasithiprasong Hospital, Northeast Thailand [20]. These dominant types were resistant to many antibiotics such as erythromycin, gentamicin, sulfamethoxazole/trimethoprim, and ciprofloxacin (Table 1). Similar to the finding of Shahsavan et al., 82% of clinical MRSA isolates in Iran were characterized as ST239, and all these strains were resistant to ciprofloxacin, erythromycin, gentamicin, sulfamethoxazole/trimethoprim, and tetracycline [32].

SCC*mec* types have been characterized in 31 MR-CoNS isolates, as shown in Table 1, and SCC*mec* type II was the dominant type of *S. haemolyticus* (62.5%). The results of this study are similar to those of Pinheiro et al., who demonstrated the association between SCC*mec* type II and *S. haemolyticus* isolated from blood cultures [33]. In contrast, Ruppe et al. demonstrated that SCC*mec* type V is preferentially associated with *S. haemolyticus* strains isolated from disparate geographical areas such as Cambodia, Algeria, Mali, and Moldova [34]. The occurrence of different SCC*mec* types in many countries might reflect the genetic background of *S. haemolyticus* strains, connected with geographical locations. SCC*mec* type III was distributed in various MRSA clones and MR-CoNS species, conforming to our results that found the distribution of SCC*mec* type III in various species such as *S. cohnii*, *S. capitis*, and *S. hominis*. Significantly, SCC*mec* type IV was associated with methicillin-resistant *S. epidermidis* (MRSE). This supported the finding of Wisplinghoff et al. that SCC*mec* type IV is distributed in many MRSE strains [35].

High prevalence of *erm*A (78.3%) and *erm*B (73.9%) genes was found in MRSA isolates, compared to the *erm*C (4.3%). The results of this study are similar to those of Lim et al. and Akpaka et al., which documented the high carriage of *erm*A and a lower prevalence of *erm*C in *S. aureus* isolates in Malaysian patients and Trinidad and Tobago patients, respectively [36, 37]. On the contrary, high prevalence of *erm*C (87.1%) was found in MR-CoNS isolates compared to the *erm*A (12.9%) and the *erm*B (12.9%) genes. Likewise, Bouchami et al. reported that the prevalence of *erm*C, *erm*A, and *erm*B of MR-CoNS isolated from bacteremic patients in oncohematology was 25.9%, 7.4%, and 7.4%, respectively [38].

We found that 26.1% of MRSA isolates carried the *qac*A/B gene. Its prevalence in the present study is higher than that in a previous report by Lu et al., who found 25 (7.8%) of the 321 MRSA isolates harboring *qac*A/B [39]. On the contrary, 70.9% of all MR-CoNS isolates in the present study carried the *qac*A/B gene. This prevalence was higher than the rate of the *qac*A/B gene carried by CoNS isolated from surgical sites (37.9%) [40], nurses (56.7%), and the general population in Hong Kong (13.5%) [23]. The increased proportion of the *qac*A/B gene in MR-CoNS indicates that hospital-acquired infections could exert selective pressure for carriage of these strains.

In summary, most of the MRSA isolates in the present study were typed as ST239-SCC*mec* type III, while different MR-CoNS species carry various SCC*mec* types. This finding provides useful information for a preventive health strategy to combat methicillin-resistant staphylococcal infections.

## Figures and Tables

**Table 1:** Molecular characterization of SCC*mec* types, disinfectant resistance genes, and antibiotic resistance genes.

Gene	MRSA, *n*=23 (%)	MR-CoNS					
		*S. haemolyticus*, *n*=18 (%)	*S. epidermidis*, *n*=3 (%)	*S. cohnii*, *n*=3 (%)	*S. capitis*, *n*=6 (%)	*S. hominis*, *n*=1 (%)	Total, *n*=31 (%)
SCC*mec* types							
I	6 (26.1)	0	0	0	3 (50.0)	0	3 (9.7)
II	1 (4.3)	10 (55.6)	0	0	0	0	10 (32.3)
III	13 (56.5)	0	0	3 (100)	1 (16.7)	1 (100)	5 (16.1)
IVa	1 (4.3)	3 (16.7)	0	0	0	0	3 (9.7)
IVb	1 (4.3)	0	0	0	0	0	0
IVc	0	0	2 (66.7)	0	0	0	2 (6.5)
IVd	0	0	0	0	0	0	0
V	1 (4.3)	0	0	0	2 (33.3)	0	2 (6.5)
Untypeable	0	5 (25.0)	1 (33.3)	0	0	0	6 (19.4)
ST239	10 (43.5)	–	–	–	–	–	–
*qac*A/B	6 (26.1)	13 (72.2)	0	3 (100)	5 (83.3)	1 (100)	22 (70.9)

Antimicrobial resistance							
*erm*A	18 (78.3)	0	1 (33.3)	0	3 (50.0)	0	4 (12.9)
*erm*B	17 (73.9)	0	1 (33.3)	0	3 (50.0)	0	4 (12.9)
*erm*C	1 (4.3)	16 (88.9)	1 (33.3)	3 (100)	6 (100)	1 (100)	27 (87.1)

## Data Availability

The data used to support the findings of this study are included within the article.

## Supplementary Materials

Supplementary Materials Supplementary Material 1: list of primers used in this study.
