# Supplementary material for: Molecular Characteristics of Methicillin-Resistant Staphylococci Clinical Isolates from a Tertiary Hospital in Northern Thailand
Source: Can J Infect Dis Med Microbiol. 2018 Nov 19;2018:8457012. doi: 10.1155/2018/8457012 (PMC6276523; doi:10.1155/2018/8457012)
Supplement: Supplementary Materials — Supplementary Material 1: list of primers used in this study. [file 8457012.f1.docx]

**Supplementary Material 1**: List of primers used in this study

| **Primers** | **Oligonucleotide sequence (5^’^-3^’^)** | **Amplicon size(bp)** | **Tm (^o^C)** | **Reference** |
| --- | --- | --- | --- | --- |
| 16S rRNA*-*F | CGAAAGCCTGACGGAGCAAC | 528 | 59.0 | [13] |
| 16S rRNA*-*R | AACCTTGCGGTCGTACTCCC |  | 59.2 |  |
| *nuc-*F(*S. aureus*) | TCGCTTGCTATGATTCTGG | 359 | 55.2 | [15] |
| *nuc-*R(*S. aureus*) | GCCAATGTTCTACCATAGC |  | 55.2 |  |
| *nuc-*F(*S. horminis*) | TACAGGGCCATTTAAAGACG | 177 | 56.3 | [41] |
| *nuc-*R(*S. hominis*) | GTTTCTGGTGTATCAACACC |  | 51.1 |  |
| *rdr-*F | AAGAGCGTGGAGAAAAGTATCAAG | 130 | 61.8 | [42] |
| *rdr-*R | TCGATACCATCAAAAAGTTGG |  | 61.8 |  |
| *gro*ESL-F | GGTCGCTTAGTCGGAACAAT | 271 | 57.8 | [43] |
| *gro*ESL-R | CACGAGCAATCTCATCACCT |  | 57.8 |  |
| *tuf-*F | CCAATGCCACAAACTCGTGA | 480 | 58.4 | [17] |
| *tuf-*R | CAGCTTCAGCGTAGTCTAATAATTTACG |  | 65.7 |  |
| *mecA*-F | TGGCTATCGTGTCACAATCG | 310 | 58 | [14] |
| *mecA*-R | GTTCTCTCATAGTATGACGTCC |  | 58 |  |
| *erm*A-F | AAGCGGTAAACCCCTCTGA | 190 | 56.7 | [45] |
| *erm*A-R | TTCGCAAATCCCTTCTCAAC |  | 55.2 |  |
| *erm*B-F | AATCGTCAATTCCTGCATGT | 142 | 55.9 | [46] |
| *erm*B-R | TAATCGTGG AATACGGGTTTG |  | 55.9 |  |
| *erm*C-F | AATCGTCAATTCCTGCATGT | 299 | 56.4 | [46] |
| *erm*C-R | TAATCGTGG AATACGGGTTTG |  | 58.2 |  |
| *qacA/B*-F | GCAGAAAGTGCAGAGTTCG | 361 | 57.3 | [23] |
| *qacA/B*-R | CCAGTCCAATCATGCCTG |  | 56.1 |  |
